# Affective Synchrony and Autonomic Coupling during Cooperation: A Hyperscanning Study

**DOI:** 10.1155/2017/3104564

**Published:** 2017-11-27

**Authors:** Maria Elide Vanutelli, Laura Gatti, Laura Angioletti, Michela Balconi

**Affiliations:** ^1^Research Unit in Affective and Social Neuroscience, Catholic University of Milan, Milan, Italy; ^2^Department of Psychology, Catholic University of Milan, Milan, Italy; ^3^Department of Philosophy, Università degli Studi di Milano, Milan, Italy

## Abstract

Previous research highlighted that during social interactions people shape each other's emotional states by resonance mechanisms and synchronized autonomic patterns. Starting from the idea that joint actions create shared emotional experiences, in the present study a social bond was experimentally induced by making subjects cooperate with each other. Participants' autonomic system activity (electrodermal: skin conductance level and response: SCL, SCR; cardiovascular indices: heart rate: HR) was continuously monitored during an attentional couple game. The cooperative motivation was induced by presenting feedback which reinforced the positive outcomes of the intersubjective exchange. 24 participants coupled in 12 dyads were recruited. Intrasubject analyses revealed higher HR in the first part of the task, connoted by increased cognitive demand and arousing social dynamic, while intersubject analysis showed increased synchrony in electrodermal activity after the feedback. Such results encourage the use of hyperscanning techniques to assess emotional coupling in ecological and real-time paradigms.

## 1. Introduction

The capacity to bond with other people has been associated with a series of positive effects for human beings, such as greater self-satisfaction [1, 2] and mental and physical well-being, including, for example, resiliency, thus reducing personal distress [3–6]. The creation of such positive relationships is thought to rely upon a bidirectional bond of affective and behavioral responses between two or more individuals [7, 8].

Empiric research highlighted that during social interactions people significantly affect and shape each other's states and behaviors [9] by basic resonance mechanisms. In fact, sharing others' emotional states can provide the observers with a somatosensory framework that facilitates understanding their intentions and actions and allows the observers to understand but also to sync with other people [10–13]. Interestingly, recent research proposed that, during social exchange, such synchronization can actually occur in the form of an alignment of behavior [14, 15] and posture [16] as well as neurophysiological [17, 18] and psychophysiological measures [19–23].

The focus on interpersonal dynamics required to adapt the experimental setting to reality [24] and move from a single-person approach to a “second person” neuroscience [25] by adopting a new paradigm, “hyperscanning,” which allows the simultaneous recording of the cortical activity from two or more participants interacting together [26] by creating “spatiotemporal maps of cerebral regions involved in the generation of the social task investigated” in a study [27, 28].

Among social interactions, cooperation is an exemplificative case of joint action that involves two or more individuals during the production of common behavioral effects [29, 30] which produces a social reward per se by involving emotional mechanisms. Previous hyperscanning approach already highlighted some patterns of neural synchronization during cooperation by EEG [31–34] or functional near-infrared spectroscopy (fNIRS) [29, 35–38]. Nonetheless, the autonomic modulations with respect to such processes still need to be clarified.

In fact, the importance given to brain-to-brain coupling led to neglecting some other important information coming, for example, from autonomic synchronization. Indeed, previous research showed that the physiological activity is related to different interpersonal processes such as empathy [39] and many other social and emotional behaviors [40, 41]. More interestingly, it has been proved that physiological synchrony may reveal how people are linked with each other [42]. Also, the acquisition of autonomic indices has some advantages, since it is more feasible than imaging and electroencephalographic (EEG) methods [24]. Unfortunately, such processes have been mostly explored in conventional single-person approach [43, 44].

For example, a widely used technique considers the response of the Sympathetic Nervous System (SNS) to detect “fight-or-flight” responses that can reveal some cues about people's emotional state, as well as about their personality [45]. It is the case of electrodermal activity (EDA) recording, which identifies modifications in skin conductivity deriving from sweat emission. It comprises a tonic component representative of the general trend, called skin conductance level (SCL), together with a phasic component indicating event-related skin conductance responses (SCR) in the form of rapid fluctuations within the tonic signal [46, 47]. The presence of a coevolution of electrodermal responses has been associated with the quality of social interactions [48].

In parallel, cardiovascular activity has been also previously used to assess physiological synchrony. As already suggested by Helm and colleagues [49] heart rate is thought, among all, to increase during states of anger, fear, and sadness [50–52]. In fact, it has been proved that fluctuations in cardiovascular response are associated with negative emotions [53–56].

Some previous studies within a developmental perspective showed that the coevolution of autonomic patterns is associated with parent-infant coregulation (see, e.g., [57, 58]). It was found that behavioral and physiological synchrony during parent-infant play covaries with infant self-regulation and cognitive and theory-of-mind abilities. For example, Feldman [57] investigated the association between biological and social rhythms by measuring sleep-wake cyclicity and heart rate variability and demonstrated their contribution to the emergence of parent-infant interactive synchrony. Interestingly, the author suggested that the temporal organization of such physiological indicators permits the infant's capacity to be part of a matched social dialogue. Indeed, by beginning with this first experience of dyadic tuning, infants learn to coconstruct optimal affective states during social interactions and to be part of complex social organizations [59, 60].

Similarly, still considering significant bonds, peripheral synchronization has been associated with couples' linkage and affective exchange. For example, Helm and colleagues [49] recorded respiration and heart rate from romantic partners across different laboratory tasks, including gazing and imitation. Results suggested that partner's heart rate and respiration could indicate shared physiological responses during interactions designed to elicit shared emotional arousal. Specifically, respiration patterns between romantic partners aligned with each other during the imitation and, especially, the gazing task, while heart rate showed associations in both tasks.

Finally, analogous regulatory processes have been found for patient-therapist interactions [61]. In this case, results showed that, during moments of high versus low skin conductance concordance, there were significantly more positive social-emotional interactions, including empathic mechanisms, for both patients and therapists.

All these data support the idea that autonomic synchrony could be indicative of various social and emotional exchange. Importantly, a study by Konvalinka and colleagues [20] highlighted that physiological synchrony is also mediated by social information in addition to synchronized behavior. In fact, they measured heart rate coherence during a social ritual and found that synchronized patterns varied according to people emotional closeness.

For what concerns cooperation in detail, few previous works considered autonomic modulation during cooperative/prosocial tasks. For example, Balconi and Bortolotti [62] showed participants different interpersonal scene types (cooperation, noncooperation, conflict, and indifference) while their autonomic responses (facial electromyography, SCR, and HR) were recorded. Results showed increased “positive” (zygomatic) facial expression and a higher autonomic activity (increased arousal, SCR, and HR) for cooperative condition, together with an increased “negative” (corrugator) facial expression and higher arousal (more SCR and HR) for conflictual conditions and reduced emotional involvement in response to noncooperative scenes, with lower SCR and HR values.

Nonetheless, no earlier work, at our knowledge, previously considered emotional coregulation and physiological linkage during a real cooperative social interaction. Thus, in the present study, a cooperative dynamic was artificially created in real-time in a way to explore how the autonomic synchronization varies according to social and emotional engagement. In detail, subjects were required to complete an attentional task together with a mate with the instruction to synchronize their responses to obtain a joint performance. A cooperative motivation was induced through the presentation of social feedback to reinforce the adoption of good common strategies and the subsequent achievement of joint outcomes. In fact, halfway through the task, they were informed about their success as a couple and their functional use of cooperative strategies.

Former available knowledge on the topic was acquired by considering previously existing couples of participants (see previous studies about mother-infant, romantic, and patient-therapist couples). In contrast, starting from the idea that cooperation creates shared, empathetic, emotional experiences [63], in this case the social bond was induced in real-time by making participants cooperate with each other. To explore these issues participants' autonomic activity was continuously monitored. Electrodermal indices, both SCL and SCR, as well as cardiovascular measures (HR) were acquired in a way to assess subjects' affective state and synchrony.

To summarize, the aim of the present study was to investigate the autonomic synchronization in dyads of participants during bond construction, which was artificially induced by administering social feedback in a way to reinforce their cooperative strategies and, consequently, their psychological bond.

Two different orders of analysis have been performed: intrasubject analysis was conducted in a way to assess participants' general physiological state during different phases of bond construction. According to previous evidence, we expected increased arousal responses, and in detail higher HR, in the first part of the task. Here, in fact, the adoption of shared strategies to synchronize with the other mate could heighten the cognitive load necessary to perform the task. Also, before the social reinforcing, the companion could be still not perceived as an ally, thus leading to less cooperative responses. On the contrary, we expected that, after receiving the social feedback about the adoption of good joint strategies, participants could begin to perceive and reinforce the couple bond, with subsequent decreased arousal, which could be framed into a more positive, close dynamic.

Then, in the second step intersubject analysis has been performed in a way to assess couples' synchrony by autonomic indices across the task. In this case, we expected an increased synchronization after the social manipulation, in line with heightened social engagement and bonding, with a subsequent increased physiological linkage and synchronicity throughout the task, as assessed by electrodermal indices (both SCL and SCR).

## 2. Material and Methods

### 2.1. Participants

24 participants, 10 females and 14 males, coupled in 12 dyads were recruited (M_age_ = 22.95, SD = 1.22). Each couple was composed of two individuals of the same sex, matched for age. They did not meet and were not familiar before the experimental session. The participants were all right-handed; they presented normal or corrected-to-normal visual acuity and gave informed written consent to participate in the study. No neurological or psychiatric pathologies were observed, based on preliminary exploration. The research was approved by the local ethics committee of the Department of Psychology, Catholic University of Milan. No payment was provided for their participation.

### 2.2. Procedure

Subjects were comfortably seated in a moderately darkened room with a computer screen positioned approximately 60 cm from their eyes. They were required to perform a simple task for sustained selective attention (modified from the original task of Balconi and Pagani [29, 64–66]). To engage subjects in the task, they were told that some cognitive attentive indices were used to evaluate their subjective skills and that these measures are usually used as a screening to test future professional career success and teamwork capabilities. Thus, the development of a joint cooperative strategy by the couple was reinforced. They were seated side-by-side but separated by a dark screen to prevent visual contact and to avoid any effect due to nonverbal behavior.

**Figure 1:**
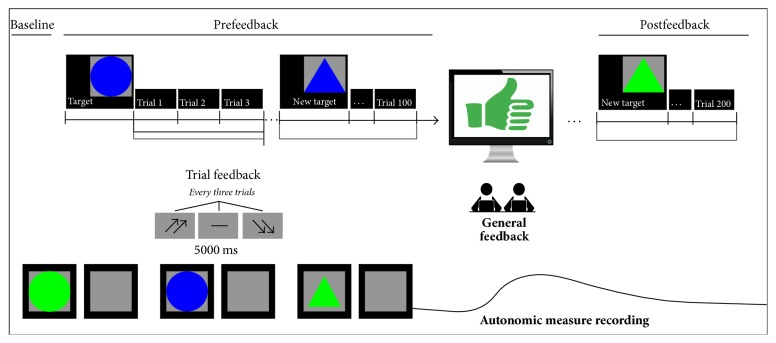
Experimental setting with autonomic measures recording.

The attentional task required to select target stimuli between nontargets, based on four different combinations of shape and color: triangles and circles and blue and green. Each target was displayed on the screen and subjects were asked to keep it in memory. Then, stimuli were presented one after another in a randomized order (see Figure 1). The target stimulus features were varied in every experimental block, composed of 25 trials. The task was composed of eight sessions (eight blocks of 25 trials each). Subjects were instructed to answer all the stimuli by pressing left/right buttons to decide between targets or nontargets. Each stimulus was presented on the screen for 500 ms, with a 300 ms interstimulus interval (ISI). Each trial was composed of three stimuli.

At the end of each trial, subjects received feedback after 5000 ms in the form of two up-arrows (high cooperation score); a dash (mean performance); or two down-arrows (low cooperation score). This feedback was shown for 5000 ms and then an intertrial interval (ITI) occurred for another 5000 ms. Thus participants constantly received an evaluation of their cooperative performance, fixed by the researcher. Besides trial-feedback, after the first four blocks (halfway of the task) subjects received a general feedback, still premanipulated, which informed subjects they had a good cooperation (temporal synchrony and paired performance: score with 87% in terms of speed and 92% in terms of accuracy). They were also encouraged to keep their performance level during the experiment. During the task, after an initial mean performance, subjects were constantly reinforced about their good cooperation by presenting the up-arrows in 70% of cases, while the dash or the down-arrows appeared in 30% of cases.

### 2.3. Autonomic Measures Recording and Analysis

Two portable Biofeedback xpert^2000^ systems with radio module MULTI (Schuhfried GmbH, Mödling, Austria) have been used for the recording of the autonomic activity. The system is capable of measuring skin conductance level and response (SCL, SCR) in *μ*S and heart rate (HR) in beats per minute (pbm). SCL was recorded with an EDA1 gold electrode using current-voltage measurement at a sampling rate of 2 kHz. The use of alternating voltage prevents polarization. The measurement resolution for SCL was 12 nS, with a sampling rate of 20 Hz. HR was measured by infrared absorption principle with a sampling rate of 500 Hz. The range of parameter was 30–200 bpm. Moreover, the mobility of the nondominant hand was monitored with an accelerometer in m/s^2^ integrated into the sender unit to ensure that recordings were not compromised by hand movements. Trials with motor artifacts were excluded from the analyses. All sensors were combined in one unit which was attached to the volar surface of the middle section of the forefinger of the nondominant hand.

## 3. Results

Two orders of analyses were performed: a first step included a general analysis (repeated measure ANOVA) about the modulation of the dependent variables (SCL, SCR, and HR) throughout the task. A second step included the calculation of intersubjects correlational indices finalized to compute the synchronization indices within each couple for each autonomic measure. Such indices were successively entered as dependent variables into different ANOVA tests, one for each autonomic measure with independent factor feedback (pre; post) and block (from 1st to 4th), to assess differences in synchrony strength across the experimental conditions. For all the ANOVA tests, the degrees of freedom have been corrected using Greenhouse–Geisser epsilon where appropriate. Post hoc comparisons (contrast analyses) were applied to the data. Bonferroni test was applied for multiple comparisons.

### 3.1. Intrasubject Analysis

A first step of analysis was intended to calculate the general trend of peripheral indices during the task by means of three repeated measures ANOVAs with feedback (2: pre, post) and block (4, each block of task) as repeated factors applied to SCL, SCR, and HR dependent variables.

**Figure 2:**
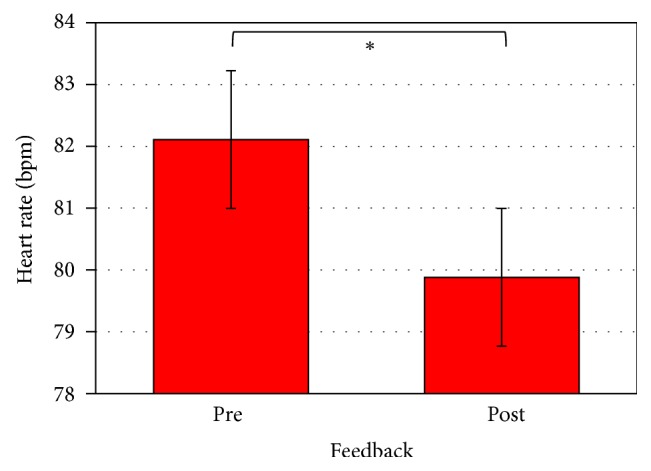
HR during pre- and postfeedback conditions. *∗* refers to statistically significant comparisons (*p* < 0.05).

The ANOVA applied to HR measures showed a significant main effect for feedback (*F*1,23 = 5.95; *p* < 0.05; *η*^2^ = 0.21) with higher HR before (M = 82.1, SD = 3.13) than after (M = 79.89, SD = 3.08) the feedback (see Figure 2). For what concerns SCL and SCR, no significant results emerged.

### 3.2. Intersubject Analysis

A second step of analysis consisted in calculating the synchronization indices by correlational coefficients applied to the data for each autonomic index (SCL, SCR, and HR), within each couple of subjects (see [67] for the procedure; see also [41, 48, 68] for different approach).

According to these indices, the subsequent third step of analysis was finalized to test the statistical significance of independent factors on these correlational indices by using repeated measures ANOVAs which included independent factor feedback (2: pre, post) and block (4).

**Figure 3:**
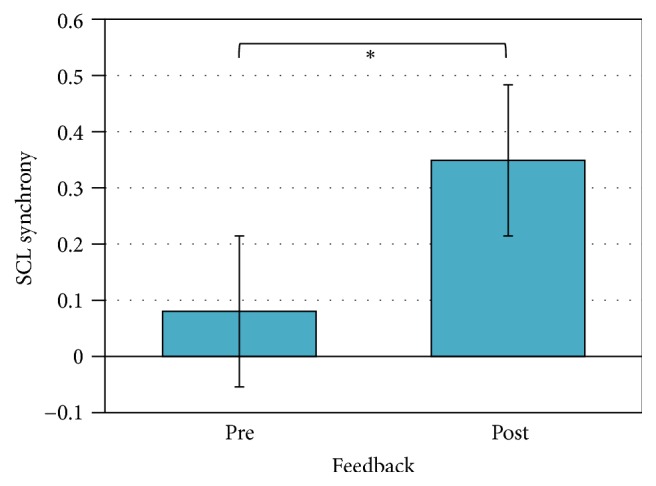
Pearson's coefficients of SCL intersubject indices as a function of feedback manipulation. *∗* refers to statistically significant comparisons (*p* < 0.05).

For what concerns HR coefficient data, no significant differences in synchrony were found. Considering SCL data, instead, a significant main effect for feedback (*F*1,11 = 9.24, *p* < 0.05, *η*^2^ = 0.46) showed increased SCL synchrony coefficients after (M = 0.35, SD = 0.12) than before (M = 0.08, SD = 0.09) the feedback (see Figure 3).

**Figure 4:**
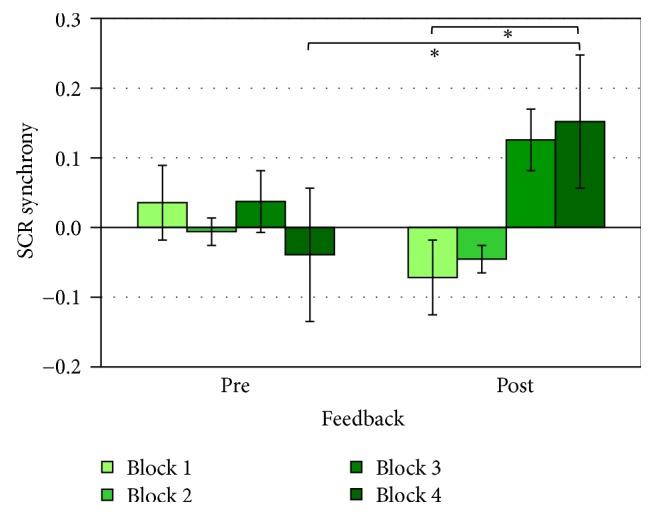
Pearson's coefficients of SCL intersubject indices as a function of feedback × block manipulation. *∗* refers to statistically significant comparisons (*p* < 0.05).

For what concerns SCR data, instead, a significant interaction effect was found for feedback × block (*F*3,33 = 3.65, *p* < 0.05, *η*^2^ = 0.25). Post hoc comparison showed that in block 8 synchrony of SCR was significantly (*p* < 0.05) higher (M = 0.15, SD = 0.04) than block 5 (M = −.07, SD = 0.05) and block 4 (M = −0.04, SD = 0.05; *p* < 0.05) (see Figure 4).

## 4. Discussion

By using a hyperscanning paradigm, the present research analyzed a joint action focusing on the autonomic response during a cooperative task which reinforced the positive outcomes of the intersubjective action. Two different orders of analysis permitted highlighting some main important effects: intrasubject analyses were conducted in a way to assess participants' general physiological state during different phases of bond construction. Then, intersubject analyses have been performed in a way to assess couples' synchrony by autonomic indices across the task. Specifically, synchrony of autonomic variables was considered during the steps of progressive reinforcing conditions in response to an attentional task.

For what concerns intrasubject analysis, higher HR was found before the feedback, together with decreased values after the social reinforce. Cardiovascular modifications usually reflect metabolic adjustment to environmental demands. Generally speaking, cardiac acceleration is related to increased stress and autonomic arousal [69]. Therefore, this result could be justified by considering two main explanations: a cognitive and an emotional one. For what concerns the cognitive one, the first part of the task could be related to higher cognitive and behavioral demand. Interestingly, previous research [70] revealed that a high cognitive load has itself the power to induce physiological arousal. For instance, Fibiger and colleagues [71] reported higher cardiac output during a mental arithmetic task. Similarly, Turner and Carroll [72] described heart rate increases during mental arithmetic and a video game. Cardiovascular responses have also been found to be sensitive to the level of difficulty in several cognitive tasks, including, for example, Raven's matrices [73], and sentence comprehension [74]. Similarly, both heart rate and general metabolic rate increased with greater cognitive load of a working memory task. Such results seem to confirm the hypothesis that the first part of the task could have been characterized by a higher level of cognitive demand in the attempt to synchronize visual, attentive, and behavioral responses. However, considering the emotional hypothesis, higher HR in the first blocks could be generated by higher arousal deriving from the social dynamic. In fact, the request to synchronize and to adopt common strategies with a stranger could be uncomfortable and stressing for participants. In this case, increased HR may be interpreted as a fight-flight response related to avoidant behaviors. In fact, it has been proved that, during a stressful condition, the presence of a stranger is associated with increased cardiovascular reactivity, while the presence of a friend can reduce it [75]. Accordingly, the HR decrease after the reinforce could be indicative of a less arousing condition, possibly attributable to a different, closer perception of the other mate.

Concerning, instead, intersubject analysis, increased synchrony in electrodermal activity was observed after the feedback. Indeed results showed heightened SCL synchronization in the second half of the task and a modulation of SCR also across blocks, with increased peripheral synchronization after the social feedback, but also an exponential increasing also within the second half. Such results suggest the presence of an increased pattern in peripheral synchronization after the social reinforcing that could signal increased engagement.

In fact, as previously discussed, research on autonomic synchrony revealed that the covariation between individuals in their physiological indices can reveal insights about the quality of their interaction [42]. For example, synchrony of electrodermal indices has been associated with couples' affective exchange [76, 77], to the quality of social interactions [48, 78], as well as to dyadic gaming experience [79] and regulatory behavior during therapy [61, 77]. More importantly, it has been considered as a key marker of social engagement [67]. Previous applications of these methods to assess the quality of interaction referred to mother-infant early interactions and psychotherapy research. For example, in a study by Ham and Tronick [77] based on a still-face paradigm, skin conductance (SC) concordance correlated with behavioral synchrony. Considering psychotherapy research, instead, a study by Marci and colleagues [61] investigated skin conductance modulation as a potential marker of therapist's empathy. Results showed that clients and therapists had significantly more positive social-emotional responses during high autonomic concordance. Thus, it is possible to assume that in the present study the social manipulation did actually enhance social engagement and bonding. In fact, as pointed out by both SCL and SCR, coregulation of autonomic activity was enhanced after the social manipulation. Also, SCR revealed an exponential increased synchronization within the second half of the task, reaching the maximum level in the last block. The present result can be explained by considering the distinct functional role of SCL and SCR measures: in fact, since SCR detects the rapid fluctuations of event-related responses, it could also be more sensitive to the trial-feedback proposed throughout the different blocks.

Considering previous hyperscanning research on cooperation with neural coupling, similar and parallel dynamics could be observed. For example, functional near-infrared spectroscopy (fNIRS) was applied to record subjects' brain activity during the same dual task performed in the present work [29]. fNIRS results revealed an increased brain activity and higher synchronization over the prefrontal cortex (PFC) after the feedback. Moreover, a significant prefrontal brain lateralization effect emerged, with the left hemisphere being more involved in the second part of the task, in line with the perception of a positive social dynamic. Such results are crucial since the involvement of prefrontal areas has been associated with social exchange, such as perspective taking and theory of mind [80], but also during the suppression of selfish behavior [81] and the commitment in significant relationships [82]. Thus, after receiving a positive feedback about the synchrony of the couple, increased connectivity emerged in areas related to empathy and bonding and, importantly, in the suppression of self-centered behaviors in favor of a common goal. The coherence between these areas was not present before the social reinforce.

However, some caution should be paid when interpreting our findings: in fact, other previous work on autonomic coupling highlighted how an increased linkage could derive from different, opposite, social, and emotional dynamics. For example, Levenson and Gottman [41] found greater physiological linkage in distressed couples, which also showed negative affect. Analogous results have also been found by Kaplan et al. [83] in two different studies where they compared physiological linkage between small groups composed of people who liked or disliked each other. Results reported significant correlations in skin conductance within the groups with “dislike” dynamics. Thus, even if our findings have been framed within a context of positive emotions and social bonding, based on our specific experimental paradigm, this issue could be better explored in future research.

## 5. Conclusions

To summarize, the present results permitted exploring autonomic coupling in dyads of subjects creating a social bond in real-time by cooperating in an attentional task presented as a couple game. Results allowed highlighting the presence of two main results, one related to a general increased arousal and HR during the first part of the paradigm, without the social reinforce, for participants considered individually. The second one deals with increased autonomic coupling during the second part, which involves the social reinforce, for participants considered within the dyad, and seems to be characterized by increased synchrony. Such results encourage the use of hyperscanning techniques to assess emotional coupling in ecological and real-time paradigms with very easy and less expensive methods.

Nonetheless, some limitations could also be addressed to the present work: first of all, the number of couples should be increased in a way to improve statistical analyses at the couple level.

In second instance, different computation methods, such as linear and nonlinear predictive models [41, 48, 68], can also be applied, especially when dealing with complex data, including multiple physiological measures. For example, by applying time-series analysis [41], it is possible to control autocorrelation problems by calculating the amount of variance of the physiological index, net to each subject's own variance. In addition, they allowed inferring if and how physiological linkage could predict affect and other measures of dyadic bonding in real life, such as marital satisfaction [41].

Moreover, a comparison condition could be added, for example, about competition, in order to explore the presence of distinct patterns of synchronization.

Finally, some behavioral measures could be introduced to help clarifying the emotional and social nature of cooperative interactions. In fact, physiological linkage per se is not sufficient to provide a complete interpretation of our findings in terms of positive/negative valence. Since such measures have already been considered in previous research on neural coupling with respect to performance and explicit, subjective variables (see, e.g., [29, 84, 85]), their introduction would be desirable in other future work on autonomic synchrony.

Also, subjective factors including participants' personality and attitudes, as well as dyadic synergy, could be considered to group dyads according to different levels of engagement and to explore the subjective attitudes to act jointly.
